# P-1494. Factors Associated with Intravenous Sodium Stibogluconate Treatment Failure in Mucocutaneous Leishmaniasis: A Case-Control Study in Cusco, Peru, 2012-2022

**DOI:** 10.1093/ofid/ofae631.1664

**Published:** 2025-01-29

**Authors:** Katherin Arando-Torres, Jesus Perez-Castilla, Fátima Concha-Velasco

**Affiliations:** Universidad Nacional de San Antonio Abad del Cusco, Cusco, Cusco, Peru; Universidad Nacional de San Antonio Abad del Cusco, Cusco, Cusco, Peru; Universidad Nacional de San Antonio Abad del Cusco, Cusco, Cusco, Peru

## Abstract

**Background:**

Mucocutaneous leishmaniasis is a neglected infectious disease primarily affecting impoverished individuals across various regions worldwide. Untimely diagnosis and treatment often result in lasting physical and psychological consequences for patients, significantly impacting their quality of life. Currently, first-line pharmacotherapy consists of pentavalent antimonials, with a treatment failure rate of over 30%. We explored factors associated with intravenous sodium stibogluconate (Sb) treatment failure in mucocutaneous leishmaniasis.

**Methods:**

This retrospective case-control study was conducted at Antonio Lorena Hospital in Cusco, Peru, from 2012 to 2022. Clinical records meeting the criteria of confirmed mucocutaneous leishmaniasis through smear, culture, PCR, or histopathology, with completion of a 30-day intravenous Sb treatment regimen, were analyzed. To calculate the sample size, proportions for independent cases and controls were used, with an Odds Ratio of 2. Moderate to severe disease was defined as more than two mucosal areas compromised with dyspnea. Cases were defined as individuals experiencing treatment failure after the initial 30-day antimonial cycle; whereas controls were those successfully cured after one 30-day antimonial cycle. Adjusted odds ratios were calculated using a logistic regression model. Ethics approval was obtained for the study.

**Results:**

A total of 124 clinical records were included, with a 49.6% intravenous Sb treatment failure rate in mucocutaneous leishmaniasis after the 30-day treatment. Of these, 75.8% (94/124) were male, 53.2% (66/124) were farmers, 17.7% (22/124) had comorbidities, 69.4% (86/124) had more than 12 months of disease, and 66.1% (82/124) had moderate to severe disease. The intravenous Sb treatment failure was associated with disease duration over 12 months (OR: 5.77, 95% CI: 2.01-16.52, p=0.001), comorbidities (OR: 21.12, 95% CI: 3.89–114.89, p< 0.001), and moderate-severe disease (OR: 2.63, 95% CI: 1.05–8.72, p=0.048).

**Conclusion:**

Our case-control study suggests that intravenous Sb treatment failure is associated with disease duration over 12 months, comorbidities, and moderate to severe disease. However, further studies are needed to elucidate this association.

**Disclosures:**

**All Authors**: No reported disclosures

